# The Effect of Cycling Through a Projection-Based Virtual Environment System on Generalized Anxiety Disorder

**DOI:** 10.3390/jcm8070973

**Published:** 2019-07-04

**Authors:** Tsai-Chiao Wang, Chia-Liang Tsai, Ta-Wei Tang, Wei-Li Wang, Kuan-Ting Lee

**Affiliations:** 1 Institute of Physical Education, Health & Leisure Studies, National Cheng Kung University, Tainan City 701, Taiwan; 2 Department of Leisure and Recreation Management, Asia University, Taichung City 413, Taiwan; 3 Institute of Innovation and Circular Economy, Asia University, Taichung City 413, Taiwan; 4 Department of Medical Research, China Medical University Hospital, Taichung City 402, Taiwan; 5 Department of Family Medicine, National Cheng Kung University Hospital, Tainan City 704, Taiwan

**Keywords:** projection-based virtual environment system, generalized anxiety disorder, cycling, virtual reality

## Abstract

Virtual reality (VR) has the potential to help clinical medicine manage generalized anxiety disorder (GAD). However, patients with GAD who use traditional head-mounted VR to cycle may cause them to feel motion sickness and fatigue. To solve this problem, a projection-based virtual environment (VE) system was built to provide GAD patients with a sense of immersion while they are cycling. This projection-based VE system allows patients with GAD to interact with the virtual environment and produce experiences similar to cycling in the outdoors. Sixty GAD patients met several screening criteria and were selected as participants. All participants were randomly assigned to one of the two 20-min conditions: (1) Observing watercolor paintings projected by the projector while engaged in cycling with a stationary bicycle; or (2) observing the scenes (i.e., forest or park) projected by the VE system and engaging in cycling with a stationary bicycle. Finally, this study confirmed that patients with GAD in the projection-based VE group exhibited higher alpha values and lower galvanic skin responses (GSR) after cycling than those cycling in the control group. These results showed that cycling in the projection-based VE group allowed the patient with GAD to achieve higher exercise intensity and lower perceived emotional stress.

## 1. Introduction

Generalized anxiety disorder (GAD) refers to situations in which patients experience persistent, excessive, and invasive concerns that daily life becomes difficult [1]. Excessive anxiety means that even if there is no threat or concern that is not proportional to the actual risk, the individual still spends a lot of time worrying about something. Unlike other anxiety disorders usually associate with specific stimuli or situations, GAD is characterized by constant and unspecific anxiety, involving a process of interacting systems that unfolds over time in continual response to a constantly changing environment [2]. GAD symptoms are associated with impairments in social, occupational or other important functional areas [1]. In addition, GAD has been highly associated with comorbid mental disorders and depression [3], which makes the treatment of GAD more challenging. More effective GAD treatments are desired. 

Virtual reality (VR) has the potential to help clinicians manage a range of symptoms associated with anxiety disorders (e.g., [4,5,6,7]). Modern information and communication technologies have made use of digital communications and interaction technologies more and more mature and complex, thus have profoundly changed the relationship between humans and nature [8,9]. One potential application of a virtual environment is to simulate a natural experience [10,11], exposing people to a virtual natural environment [12]. Virtual nature exposure provides a soothing and awesome experience to the user through sensory stimulation, giving them the benefits of being in a comfortable space [13]. For people with depression, using an immersive virtual reality can let them realize the vision of nature and even experience the restoration effect [14]. The current new virtual environment technology makes it possible to provide a high degree of immersion and presence, which may make people experiencing high stress loads feel that they have escaped from the surrounding environment [12].

However, current VR technology requires users to wear a head-mounted display helmet to create a sense of space and depth. VR is usually delivered via a head-mounted display which tracks the users’ head-movements and allows for real-time updating of the scenes they can see [15]. There are several virtual reality exposure therapy (VRET) meta-analyses which have shown that VRET caused a significant reduction in anxiety-related symptoms (i.e., [16,17]). These studies also have confirmed that VR has been effective for generalized anxiety disorder [16,17,18]. However, the current literature on VR has rarely explored the contribution of using projection-based VR to promote exercise for the treatment of GAD. Exercise is considered to be an effective treatment for anxiety and depression. In many cases, exercise therapy can be tailored to patients to help them reduce the symptoms of anxiety and/or depression. Cycling with stationary bicycles in VR allows individuals to schedule their cycling schedules according to their own time and allows patients to adjust and improve their exercise intensity according to their physical condition [19,20]. Cycling is a sport that is worth encouraging for GAD patients. Previous studies have confirmed that cycling can increase positive emotions (i.e., [19,21]) and improve brain wave patterns (e.g., less spontaneous neuronal potential) [21].

By measuring changes in the reflex potential generated by the activity of nerve cells in the brain, the current physiological response status of the individual can be known. The alpha wave in the brainwave represents a person’s most sober, quiet, stable, and focused state of mind. The large amplitude of the alpha wave can indicate that the individual is currently in a state of waking and relaxing. The higher the value of the alpha wave is, the more awake and relaxed the individual is; in other words, a person’s mind is conscious and lucid, while the body is relaxed. A small amplitude of the alpha wave indicates that the individual is currently in a state of tension and uneasiness [22]. 

In addition, currently available VR technology requires the use of head-mounted display helmets to create a sense of space and depth. However, this is a challenge for patients who are not comfortable exercising in the outdoors as exercising with a head-mounted display helmet generates safety concerns. To solve this problem, the authors constructed a new way to provide VR in a clinical setting through a projection-based virtual environment (VE) system. This technology can provide patients with a sense of immersion while they exercise indoors. The projection-based VE system allows users to interact with the virtual environment and provide them with a cycling experience similar to cycling outdoors. The projection-based VE system is not similar to the head-mounted VR [23]. Individuals using the projection-based VE systems to engage in exercise are less likely to experience motion sickness and fatigue which commonly results from head-mounted VR [23,24,25]. The projection-based VE system can elicit realistic emotional responses, providing a novel clinical opportunity for exposure and improvement in athletic performances, as well as the evaluation of treatment effects using continuous tracking of anxiety and stress obtained during projection-based VE exposure [26]. Thus, the authors hypothesized that immersive virtual environment-based cycling could facilitate cycling behavior and improve the cycling experience of patients with GAD compared with a non-immersive system. Therefore, this randomized controlled trial was conducted to determine whether projection-based VE systems reduced the perceived stress, improved positive mood and athletic performance.

## 2. Experimental Section

### 2.1. Participants

For older adults, regular exercise is an effective method to slow down functional deterioration caused by aging [27,28]. Recreational sports activities have a positive influence on the health of older adults, including a reduced mortality rate [29], delayed aging and lowered medical costs [30]. Therefore, the authors chose people aged 50–75 as participants. In addition, cognitive diseases might affect the outcome of GAD. To prevent participants from affecting the results of the study because of other cognitive conditions (i.e., Alzheimer’s disease), the authors first performed the mini mental state examination (MMSE) tests to screen the participants. Tombaugh & McIntyre (1992) [31] demonstrated that the MMSE have validity for assessment of the degree of cognitive impairment. MMSE could provide a brief screening test that quantitatively assesses the severity of cognitive impairment.

To avoid possible interference of health conditions (e.g., obesity), sedentary behavior and cycling competence with the experimental outcomes, several criteria were chosen to obtain a sample of noncycling, healthy, and physically active adults. The study selected participants by several criteria: (a) Initial diagnosis of GAD; (b) aged between 50 and 75 years; and (c) never experienced VR before; (d) their scores of the mini mental state examination (MMSE) achieved 27 or higher; (e) they were at normal weight according to the body mass index (BMI) standard defined by the Department of Health (DOH) in Taiwan as 18.5 ≤ BMI ≥ 24 kg/m^2^; and (f) they did not practice cycling, either recreationally or competitively. The study also adopted the following exclusion criteria, including: (a) Suffering from anxiety disorders other than GAD; (b) having claustrophobia because the experiment was performed in an immersive wrap-around system, which is quite a narrow area. The authors contacted a total of 68 participants, and 8 participants were excluded due to the presence of other anxiety disorders. Several subjects were unable to adapt to the experimental environment, therefore, they quit halfway. The final sample included a total of 60 participants who all met the criteria. 

The assessment of generalized anxiety disorder was performed using generalized anxiety disorder 7–item (GAD-7) to screen participants. GAD-7 is a seven-item questionnaire that measures the severity of GAD [32]. The subjects were asked to assess the feeling they encountered in life: “Over the last 2 weeks, how often have you been bothered by following 7 items?” Each item has a four-point Likert scale (0 = not at all sure, 1 = several days, 2 = over half the days, and 3 = nearly every day). The total scores range from 0 to 21 and represent four different severity levels: Without symptoms (scores range from 0 to 4); mild anxiety symptoms (scores range from 5 to 9); moderate anxiety symptoms (scores range from 10 to 14) or severe anxiety symptoms (scores range from 15 to 21). The GAD-7 has high sensitivity and good specificity for detecting GAD [33]. A higher the total score represents more serious level of GAD. The participants were diagnosed with generalized anxiety disorder if they met the diagnostic criteria of GAD-7. Among the 60 participants, 47 participants had a GAD score of 10 or more, which was a moderate and severe generalized level of depression. Further, 13 participants had a mild level of GAD.

### 2.2. Projection-Based Virtual Environment System

**Figure 1:**
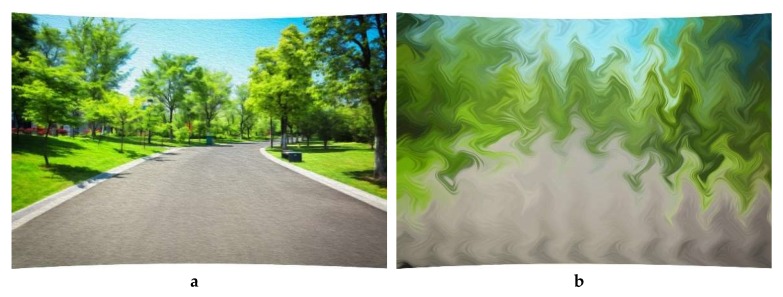
Simulated images for experiment. (**a**) Projection-based VE condition; (**b**) Control condition.

This virtual environment projection system presents a large screen of 270 degrees, which allows the subject to feel the sense of immersion. This system uses wireless serial technology to synchronize the cycling speed with the speed of movement in the virtual environment. To prevent the sound from affecting the experimental results, no sound was transmitted to the subject during the experiment. The authors are applying for patents about the whole and part of the projection-based virtual environment system. The simulated images under VE and control conditions were shown in Figure 1.

### 2.3. Preparation

This experiment required participants to concentrate for a long time. Therefore, the authors reminded the subjects to avoid activities that would interfere with physiological indicators within 24 h prior to the specified experimental time, such as drinking caffeinated beverages, taking medication, or staying up late. Furthermore, to better measure the brain wave of the subject, the authors recommended that the participant wash their hair before the experiment and avoid wiping hair care products such as hair gel or hair wax.

### 2.4. Processes

This study recruited subjects through hospitals and the National Cheng Kung University. The authors first contacted the participants by phone or face to face and asked them to fill out the GAD assessment scale. After that, a medical doctor evaluated the results of the questionnaire to assess whether the subject had generalized anxiety disorder. If the doctor found that the participant’s anxiety was caused by other diseases, the participant was excluded. 

This study was carried out in accordance with the recommendations of the National Cheng Kung University’s Ethics Committee’ with written informed consent from all subjects. All 60 healthy volunteers gave written informed consent to participate in the study in accordance with the Declaration of Helsinki. The study was approved by the National Cheng Kung University’s Ethics Committee. The participants did not include minors or people with physical disabilities.

### 2.5. Design

All participants were randomly assigned to one of the two experimental groups, according to gender and physical activity level. All participants were randomly assigned to one of the two 20-min conditions: (1) On a stationary bicycle, observing watercolor painting images projected by the projector, and engaged in cycling; or (2) on a stationary bicycle, observing the scenes in the interactive virtual environment system and engage in cycling. Through this interactive virtual environment system, the participants felt that they were cycling in a forest or park. Throughout the experiment, data was collected on the participants’ brain waves and heart rates. The data from the brain waves and heart rates were used to perform a series of repeated measure analyses to test the hypotheses. 

A polar optical heart rate sensor was worn inside the participant’s arm to monitor the heart rate of the participant. The participants were asked to exercise at a maximum intensity of 50–60% maximum heart rate (HRmax). If the above heart rate values did not meet the 50–60% maximum heart rate, the participants were asked to increase or decrease their cycling speed. All experimental procedures were tested in the virtual environment laboratory of National Cheng Kung University. The experimental environment was controlled to room temperature 24–25 °C and relative humidity between 50–60%. 

The descriptive statistics for each group are shown in Table 1. The results of t-test analysis showed no significant differences in demographic variables between groups at the beginning of the experiment for age, *T* value = 0.3, *p* = 0.76; and BMI, *T* value = 0.08, *p* = 0.99; and MMSE, *T* value = 0.12, *p* = 0.90.

### 2.6. Data Collection

Stress can be measured through a variety of physiological signals, among which are galvanic skin response (GSR), heart rate (HR) and electroencephalogram (EEG). The ProComp Infiniti biofeedback system (Thought Technology Ltd., Montreal, QC, Canada) was used for data acquisition. The data, GSR, HR and EEG signals, were continuously recorded through biosensors placed on the participant. GSR is a simple, sensitive, and reproducible method of measuring the resistance of the skin gland as a marker for sympathetic nervous activity to present a reaction where the electrical conductivity of the skin is produced by the stimulations [34,35]. When an individual is sleeping or resting, the value of the GSR is lower than the average. When an individual experiences emotions such as stress or anger, the value of the GSR increases immediately [34,36,37].

The varying activity of neurons in the brain causes fluctuations in the voltage potential along the scalp that can be measured with an electroencephalogram (EEG) [38]. Previous research has identified a number of brain wave frequency bands from EEG data which included alpha (α) waves. Alpha waves can typically be observed when an individual is in a relaxed state. The higher the value of the alpha wave is, the more awake and relaxed the individual is. The EEG signals, sampled at 256 Hz, were recorded from five channels (FP1, FP2, T3, T4 and Pz) and placed on each the participant’s scalp according to the international 10–20 system. Before virtual exposure, two and a half minutes of resting EEG activity were recorded. Subsequently, cycling in the virtual environment started. After cycling, the resting state EEG was also recorded for two and a half minutes immediately. During EEG recording processes, the participants were asked to take a relaxed sitting position on the chair, close their eyes and avoid any movements.

### 2.7. Pre-Process

Raw EEG data is usually a mixture of several factors, including brain activity, blinking, muscle activity, and environmental noise. Many noises that are present in the EEG signals can be removed using simple filters. To analyze the peripheral signals quantitatively, pre-process is required to eliminate environmental noises by applying filters. The peripheral signals were filtered by a moving average filter to remove noise. The data was filtered using a band pass filter in the frequency band of 0.2~35 Hz. This band was selected because the frequency intervals of interest in EEG was the alpha wave (8–13 Hz). 

## 3. Results

Regarding the 68 GAD participants, 1 participant met the exclusion criteria for having claustrophobia, and 7 participants met the exclusion criteria for suffering from other anxiety disorders. Thus, total 8 participants were excluded from the experiment. The remaining 60 participants were randomly assigned to one of the two groups, one was the projection based VE system and the other was the control group. There were 30 participants in each group. There were no significant differences in the characteristics of the participants (Table 1), experimental equipment and experimental environment used by the participants between the two groups.

There was no significant difference between the two groups with respect to the baseline alpha values before the intervention (projection-based VE group: 3.95 (2.43–5.47) versus the control group: 3.72 (2.34–5.10); *p* > 0.05, Table 2). There was also no significant difference in the alpha values of the control group between pre- and post-exercise (*t* = 0.11; *p* = 0.092). However, the alpha values of the projection-based VE group were significantly higher than the control group after the intervention (projection-based VE group: 6.53 (1.72–11.35) versus the control group: 4.05 (2.62–5.48); *p* < 0.01, Table 2).

During the cycling exercise, GSR increased indicating sympathetic activation. The entire cycling exercise time was 20 min. No participant reported any discomfort throughout the process of the experiment. In the projection-based VE group, the measured GSR value after 20 min exercise was significantly higher than baseline value (*t* = 3.05; *p* < 0.01). Furthermore, in Table 2, a greater number of generalized anxiety disorder patients in the projection-based VE group showed lower GSR (*t* = 2.2; *p* = 0.03) than in the control group. However, there was no significant difference in the HR values between the projection-based VE group and the control group (*t* = 1.3; *p* = 0.20). In addition, in the control group, the participants with GAD in the control group showed higher HR (*t* = 2.25; *p* = 0.037) and GSR (*t* = 3.05; *p* = 0.007) after cycling than before cycling (*p* = 0.02). 

## 4. Discussion

People with GAD are often in an anxious state. Therefore, it is important to have a relaxed response through exercise. This study is the first clinical study to explore and demonstrate that virtual environments can reduce anxiety experienced by people with GAD. The purpose of this study is not only to measure the effectiveness of VE interventions, but also to determine whether using VE to present images of forests or parks during cycling can reduce anxiety and improve athletic performance. Frumkin et al. (2017) [12] called for future research to explore how natural technology can expand or even change the way humans come into contact with nature. This study not only responded to their recommendations, but further confirmed that virtual environments may be a way to improve generalized anxiety disorder. The authors developed a wrap-around virtual environment as a tool for virtual environmental therapy that allowed GAD patients to engage in cycling in the virtual environment.

VE exposure could be considered an alternative to traditional exposure because it provides an opportunity for an anxious patient to engage in exercise similar to an outdoor environment without having to interact with others. This study demonstrated that the cycling experience in VE could effectively improve the alpha waves in patients with generalized anxiety disorder. After the entire process of experiment, the alpha values of participants cycling in the projection-based VE group were higher than those cycling in the control group. In addition, participants cycling in the VE system could obtain higher GSR compared to the baseline. Participants cycling in front of watercolor paintings could obtain higher GSR than participants cycling in the VE system. Those results imply that the subjects who rode in the VE environment did not feel too much pressure and the VE environment let them rode in a more pleasant mood. These results might be due to the VE presented immersive images of forests or parks that can make these GAD patients feel relaxed. These research findings are similar to Thompson et al. (2001) [39]. They found that compared with exercising indoors, exercising in natural environments was associated with greater feelings of revitalization and positive engagement, and with lower tension and depression. Furthermore, this study also confirmed that projection-based VE cycling raised the heart rate of participants compared to the baseline. However, the HRs were not different between the two conditions. Those results showed that cycling in the wrap-around virtual environment allowed the participants with GAD to achieve a similar heart rate but perceived lower emotional stress than cycling in front of watercolor painting, as each of the participants of the two groups measured heart rate after 20 min of cycling. Therefore, the same cycling period resulted in the same level of heart rate. In sum, virtual immersion in simulated outdoor environments could enhance the potential efficacy of exercise in GAD. The results suggested that nature-based VE might further enhance the benefits of exercise in some subjects with GAD.

As GAD brings several negative effects to patients in many living conditions [40], it is quite difficult to create a scenario of VE that allows people with GAD to relax. To make patients with GAD feel relaxed, the scenarios of VE must be able to reduce many different personalization concerns experienced by patients with GAD. Given the characteristics of patients with GAD, VE-based treatments can focus on the extent to which GAD patients can reduce their concerns in specific settings (e.g., forests or parks). The anxiety of patients with GAD can be relieved by exercise in forest or park situations. The results imply that the landscapes of VE need to be realistic and relevant to the patient’s needs and preferences. Anxiety disorders are similar to GAD, but the driving force for anxiety disorders varies from patient to patient, making it difficult to develop a comprehensive VE program that meets the needs of all patients with anxiety disorders. In addition, many patients with GAD have many behaviors similar to anxiety-related behavior, and thus enhance the barriers to construct a VE system which may help GAD patients to engage in exercise. Therefore, the authors call for more research to explore the effects of virtual environments or virtual reality systems on helping patients with GAD to engage in exercise.

There are several limitations in this research. First, this study only compared the differences between projection-based VE group and a control group for GAD patients and their exercise performance, but did not compare the differences between the projection-based VE and the actual environment for GAD patients and their exercise performance. Future research can compare the effects of the differences between the two on emotion and exercise outcomes. Second, this study only explored the stress relaxation effect of a single exercise on GAD patients but did not validate the effects of long-term exercise. Future research can explore the benefits of cycling for GAD patients in the projection-based VE, such as improving mood, reducing stress, and improving athletic performance. Third, this study used cross-sectional data to explore the hypothesized relationships. To avoid common method variation, future studies may employ a longitudinal design to collect data for exploring the process by which projection-based VE producing actual cycling behaviors can achieve higher exercise intensity and reduce anxiety perceived by GAD patients.

## 5. Conclusions

The projection-based VE treatment could serve as a visual guide for practicing relaxation or cycling exercises. The projection-based VE system creates a comfortable exercise environment that allows patients with GAD to engage in cycling while relaxing. The results of the study demonstrated that the alpha waves could be aroused by cycling in virtual nature in a short time (i.e., 20 min). Cycling for 20 min in a virtual environment (i.e., parks and forests) was enough to arouse the relaxation of GAD participants and allow them to achieve exercise outcomes. Thus, to create a relaxing mood, patients with GAD can cycle in a virtual environment for 20 min, which may help them reduce the stress they feel. In addition, through projection-based VE systems, cycling can cause patients with GAD to reduce the perceived stress, improve positive mood (i.e., relax) and exercise outcomes. Cycling in an immersive projection-based VE system may help patients with generalized anxiety disorder develop regular exercise habits and reduce their anxiety symptoms. Furthermore, exercise in an indoor virtual environment allows the patient to develop and maintain regular exercise habits, and does not reduce the willingness to exercise because of rain, serious air pollution, or a lack of natural landscapes.

## Figures and Tables

**Table 1:** The characteristics of the participants.

Variables	Projection-based VE Group (*n* = 30)	Control Group (*n* = 30)	*p* Value
Age (year)	58.72 (50–70)	59.32 (50–72)	0.76
Height (cm)	1.62 (1.5–1.79)	1.58 (1.45–1.78)	0.11
Weight (kg)	62.66 (46–92)	58.79 (44.6–82)	0.21
Gender (M/F)	12/18	13/17	0.45
BMI (kg/m^2^)	23.52 (18.83–32.44)	23.52 (17.53–32.24)	0.99
MMSE	28.87 (26–30)	28.83 (25–30)	0.90

Note: BMI: body mass index; MMSE: mini-mental state examination; VE: virtual environment

**Table 2:** Comparison of alpha value, heart rate (HR) and galvanic skin response (GSR) between the projection-based VE group and the control group.

Variables	Projection-based VE Group (*n* = 30)	Control Group (*n* = 30)	*p* Value
Alpha value-before cycling (μV)	3.95 (2.43–5.47)	3.72 (2.34–5.10)	0.61
HR-before cycling (count/minute)	75.22 (67.66–82.78)	78.92 (72.67–85.17)	0.15
GSR-before cycling (micromhos)	0.63 (0.12–1.14)	0.70 (0.49–1.11)	0.13
Alpha value-after cycling (μV)	6.53 (1.72–11.35)	4.05 (2.62–5.48)	0.01
HR-after cycling (count/minute)	89.20 (78.11–100.29)	89.05 (81.71–96.39)	0.20
GSR-after cycling (micromhos)	0.81 (0.19–1.33)	1.1 (0.59–2.65)	0.03

Note: HR: heart rate; GSR: galvanic skin response.
